# Celebration of the Birth-Day of Pinel at the Stale Lunatic Asylum

**Published:** 1846-06-01

**Authors:** 


*Celebration of the Birth-Day of* Pinel *at the State Lunatic Asylum.*—The Utica Gazette contains an account of a celebration of the birth-day of the illustrious *Pinel*, at the Lunatic Asylum in that city. A hymn, written by Judge Bacon, of Utica, for the occasion, was sung by the choir of the Institution; prayers were offered by the Chaplain; and an eulogium on on the character of Pinel pronounced by Dr. Maltbie, one of the patients at the Asylum. The Gazette says that the exhibition was unique and interesting.

